# Neuroimaging of Koolen-De Vries Syndrome: A Rare Genetic Disorder

**DOI:** 10.7759/cureus.79194

**Published:** 2025-02-17

**Authors:** Karis Houser, Sara Esteves, Michael S Kuwabara

**Affiliations:** 1 Radiology, Phoenix Children's Hospital, Phoenix, USA; 2 Radiology, Creighton University School of Medicine, Phoenix, USA

**Keywords:** autosomal dominant genetic disorder, chromosome deletion, koolen-de vries syndrome, neurodevelopmental disorder, pediatric neuroimaging

## Abstract

Koolen-de Vries syndrome (KdVS) is a rare neurodevelopmental genetic disorder involving multiple organ systems. It is characterized by several abnormal neurological, behavioral, and skeletal features. This case describes a 12-year-old male who had been previously diagnosed with Koolen-de Vries syndrome and has a complex medical history. The patient exhibits many complications of KdVS, such as congenital heart disease, epilepsy, cryptorchidism, and cardiomegaly. Neuroimaging displayed a dysplastic corpus callosum, colpocephaly with mild lateral and third ventriculomegaly, and an ovoid dysmorphic appearance of the hippocampal formations. This case report serves as an educational exhibit for the neuroimaging findings and clinical features of KdVS to raise awareness of its presentation.

## Introduction

Koolen de-Vries Syndrome (KdVS) is a rare autosomal dominant genetic disorder caused by a deletion at chromosome 17q21.31 [1]. KdVS is characterized by several symptoms, including developmental delay, neonatal/childhood hypotonia, congenital malformations, dysmorphic skeletal structures, and behavioral features [1-5]. Most individuals with KdVS experience an intellectual incapacity accompanied by psychomotor and speech/language delays [1,2]. Additionally, KdVS has a notable phenotypic presentation. Hearing impairment in KdVS cases is occasional, and when it does occur, it most commonly involves conduction issues. Congenital heart defects such as atrial septal defect (ASD) and scoliosis are less common features. Cryptorchidism and skin-pigmentation abnormalities are sometimes present [1,4,5]. Epilepsy and brain malformations are observed in 80% of cases [1,3]. This patient's neuroimaging displayed distinctive KdVS features, including a dysplastic corpus callosum, colpocephaly with mild lateral and third ventriculomegaly, and an ovoid, dysmorphic appearance of the hippocampal formations.

## Case presentation

A 12-year-old male presented to the radiology department with a complex medical history. In early childhood, he was diagnosed with atrial septal defect (ASD), ventricular septal defect (VSD), complex febrile seizures, chronic lung disease, partial agenesis of the corpus callosum, esotropia, cryptorchidism, and developmental delay. The patient began a genetic workup at one year old for Noonan syndrome and cardiofaciocutaneous syndrome due to symptoms of developmental delay, mild hypotonia, complex febrile seizures, multiple congenital anomalies, and dysmorphic features most consistent with these diagnoses. This included a normal renal ultrasound and DNA testing with no mutations identified. He was continually followed by neurology, genetics, and developmental pediatrics for intellectual delay, communication deficit, disruptive behavior, feeding problems, sleeping problems, sensory processing challenges, and mild hypotonia with substantial functional limitations in activities of daily living. While improvement was noted following enrollment in preschool and additional therapies, his diagnosis included encephalopathy, possibly due to Noonan syndrome consisting of global developmental delays, communication disorder, generalized hypotonia, disruptive behaviors, and feeding disorder with dysphagia and aspiration. At five years old, he underwent bovine pericardial patch closure of the large secundum ASD. On follow-up with a developmental pediatrician, they resolved previous concerns about disruptive behaviors at home and lack of classroom participation. The patient was doing well, although academic progress remained slow. The same year, at five years old, he was diagnosed with Koolen-de Vries syndrome due to heterozygous de novo mutation, c.611dupG, causing an amino acid change p.Met205TyrfsX9 variant in the lysine acetyltransferase 8 regulatory non-specific lethal complex subunit 1 (KANSL1) gene.

At eight years old, the patient's parent reported nocturnal seizures, prompting the continuation of his anti-epileptic medications that had been previously prescribed. He was also noted to have behavioral changes. He was referred to speech, occupational, physical, and feeding therapies, as well as advised to continue to follow up with an outpatient neurologist.

**Figure 1:**
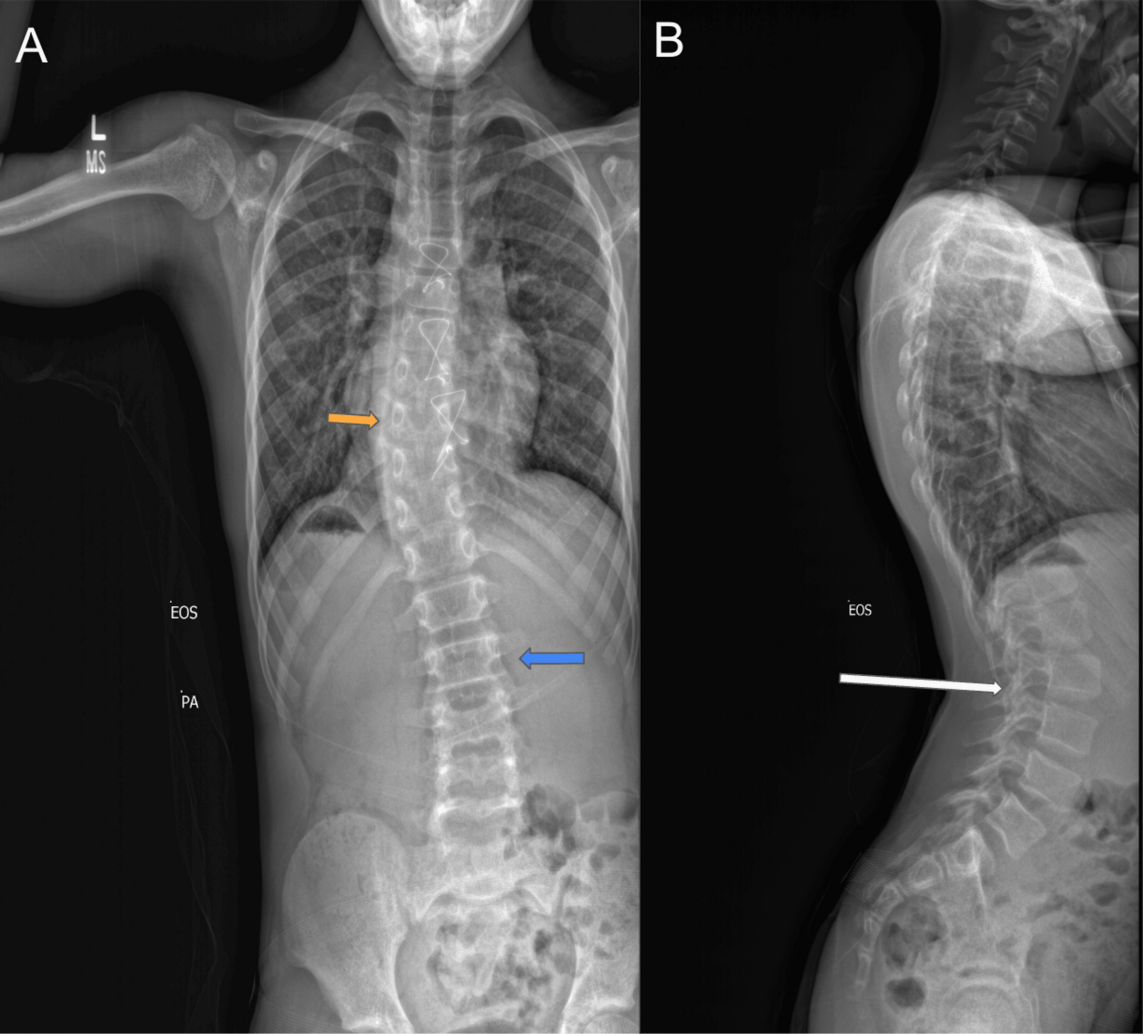
X-ray of the spine X-ray of the spine demonstrated moderate dextroconvex scoliosis of the thoracic spine, apex at T9 (A: orange arrow), mild levoconvex curvature of the lumbar spine (A: blue arrow), and exaggerated lumbar lordosis (B: white arrow). T9 - ninth thoracic vertebra

At 12 years old, on presentation to the radiology department, he was diagnosed with adolescent idiopathic scoliosis from an X-ray of the spine, which demonstrated moderate dextroconvex scoliosis of the thoracic spine, apex at T9, mild levoconvex curvature of the lumbar spine (Figure 1A), and exaggerated lumbar lordosis (Figure 1B). Later that year, he was seen by an ear, nose, and throat (ENT) specialist for hearing loss. A hearing aid was subsequently recommended for his left ear.

**Figure 2:**
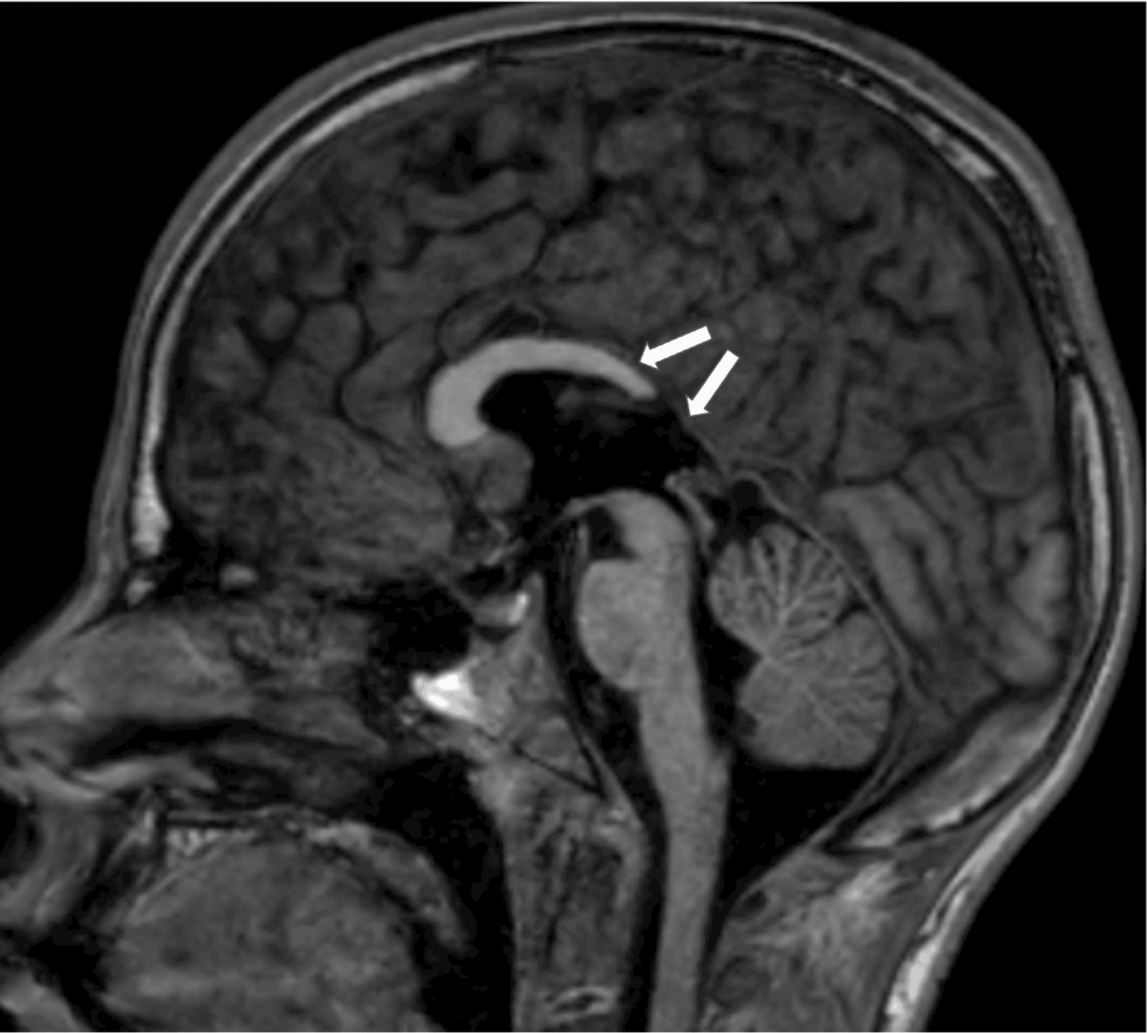
Sagittal T1-weighted MRI of the brain without contrast shows dysgenesis of the corpus callosum and agenesis of the dorsal body and splenium (white arrows) T1 - longitudinal relaxation time

**Figure 3:**
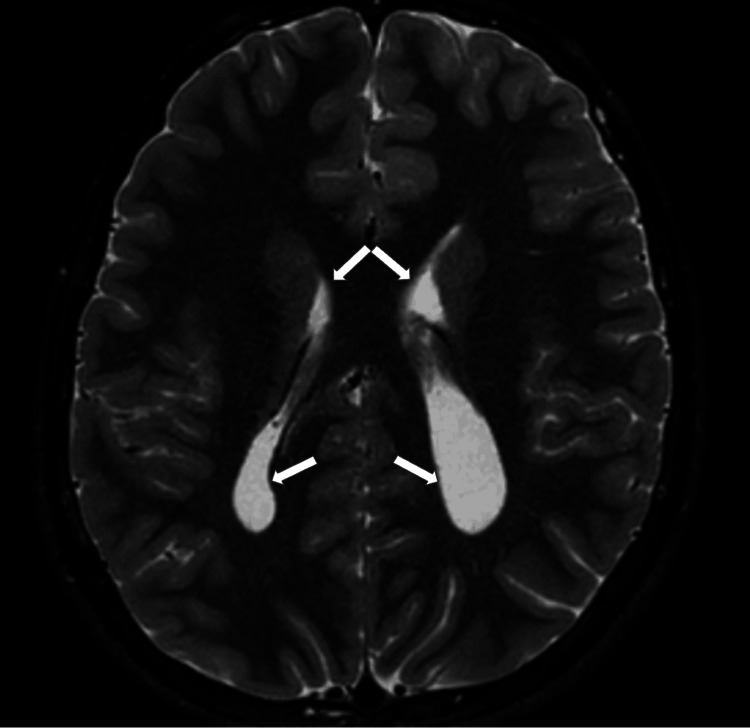
Axial T2-weighted MRI of the brain without contrast shows colpocephaly with mild ventriculomegaly (white arrows) T2 - transverse relaxation time

**Figure 4:**
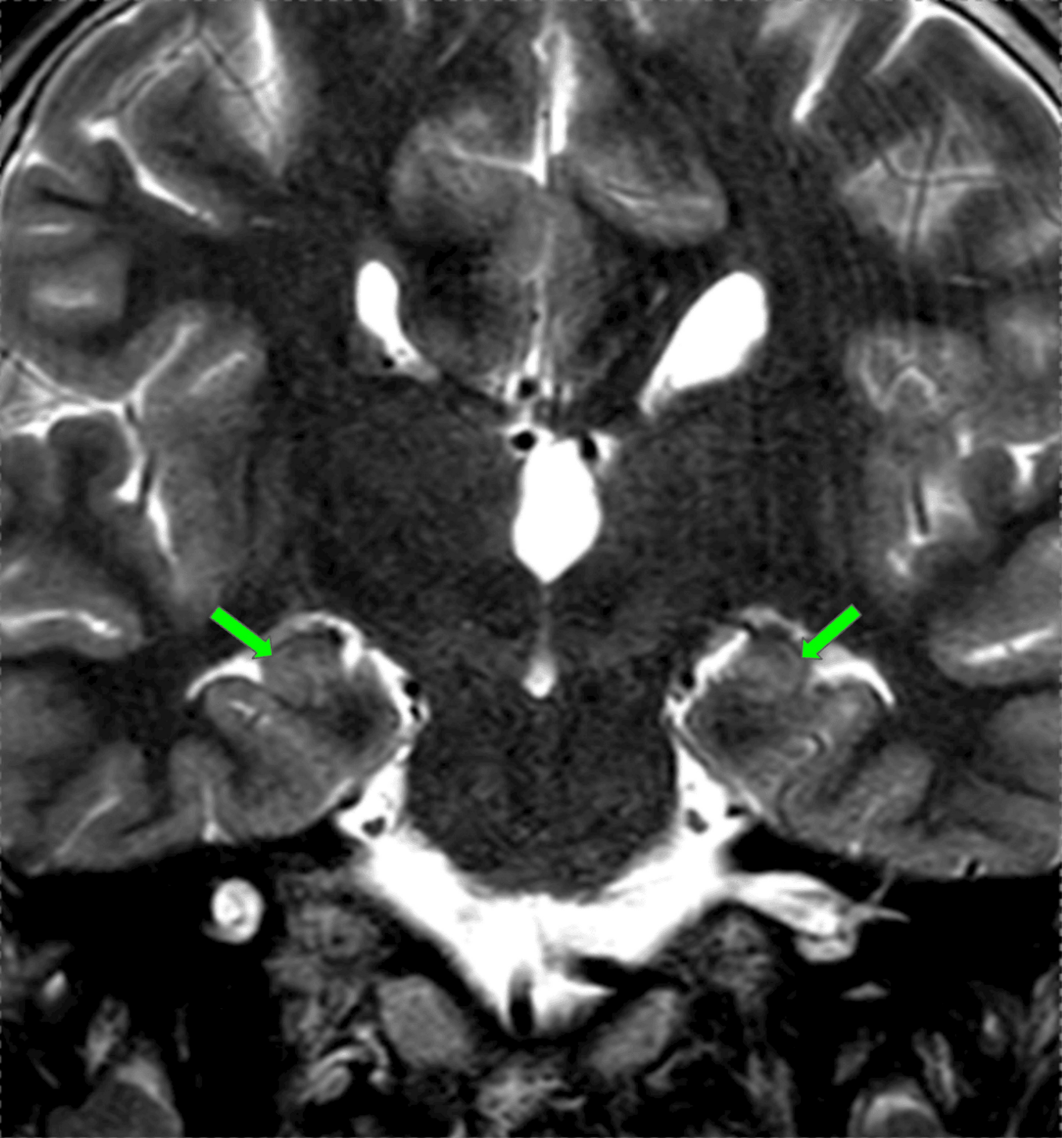
Coronal T2-weighted MRI of the brain without contrast demonstrates dysplasia, malrotation, and an ovoid shape of the hippocampi bilaterally (green arrows) T2 - transverse relaxation time

**Figure 5:**
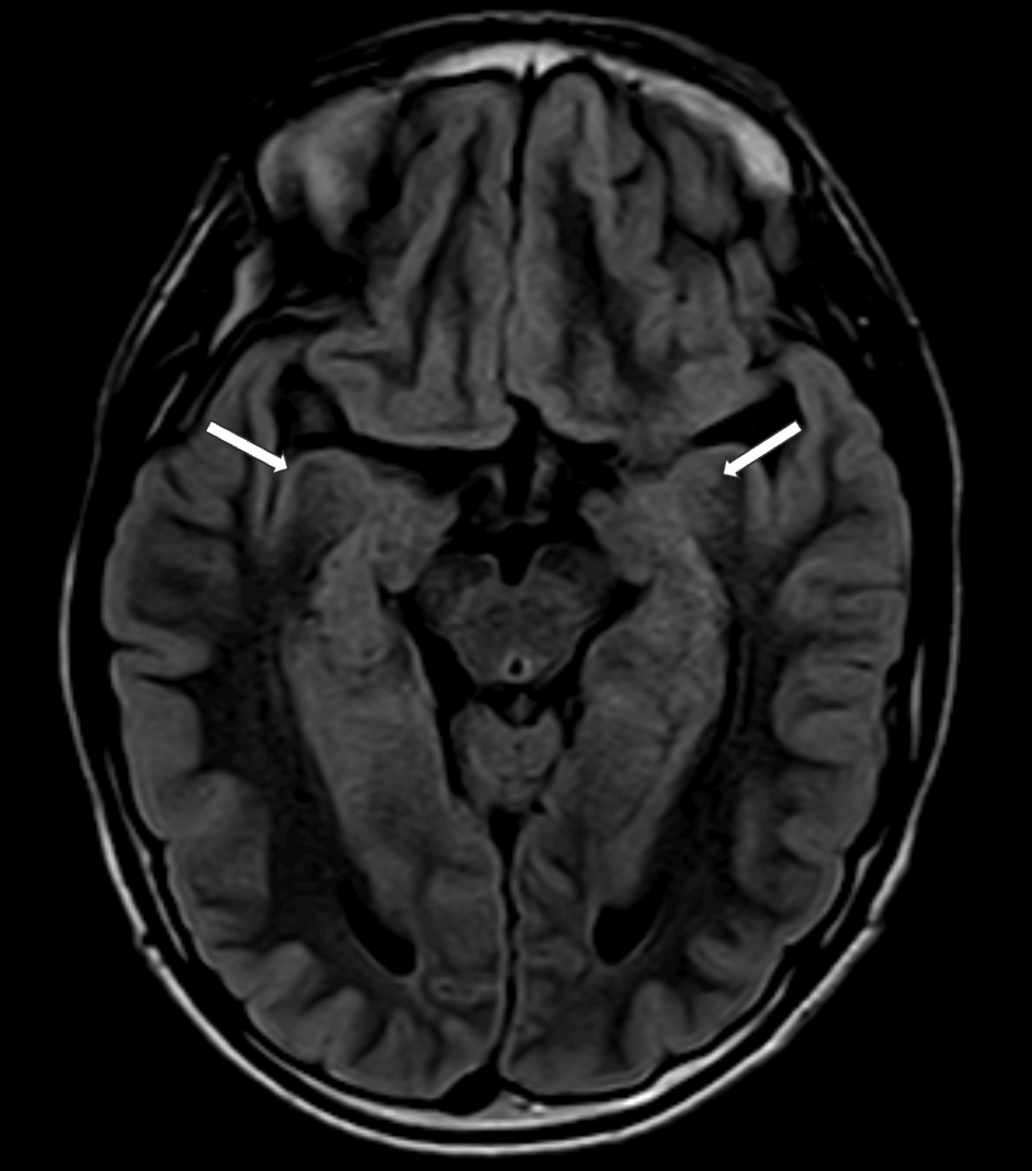
Axial-FLAIR MRI of the brain without contrast through the level of the hippocampal formations as companion to Figure 4 (white arrows) FLAIR - fluid-attenuated inversion recovery

**Figure 6:**
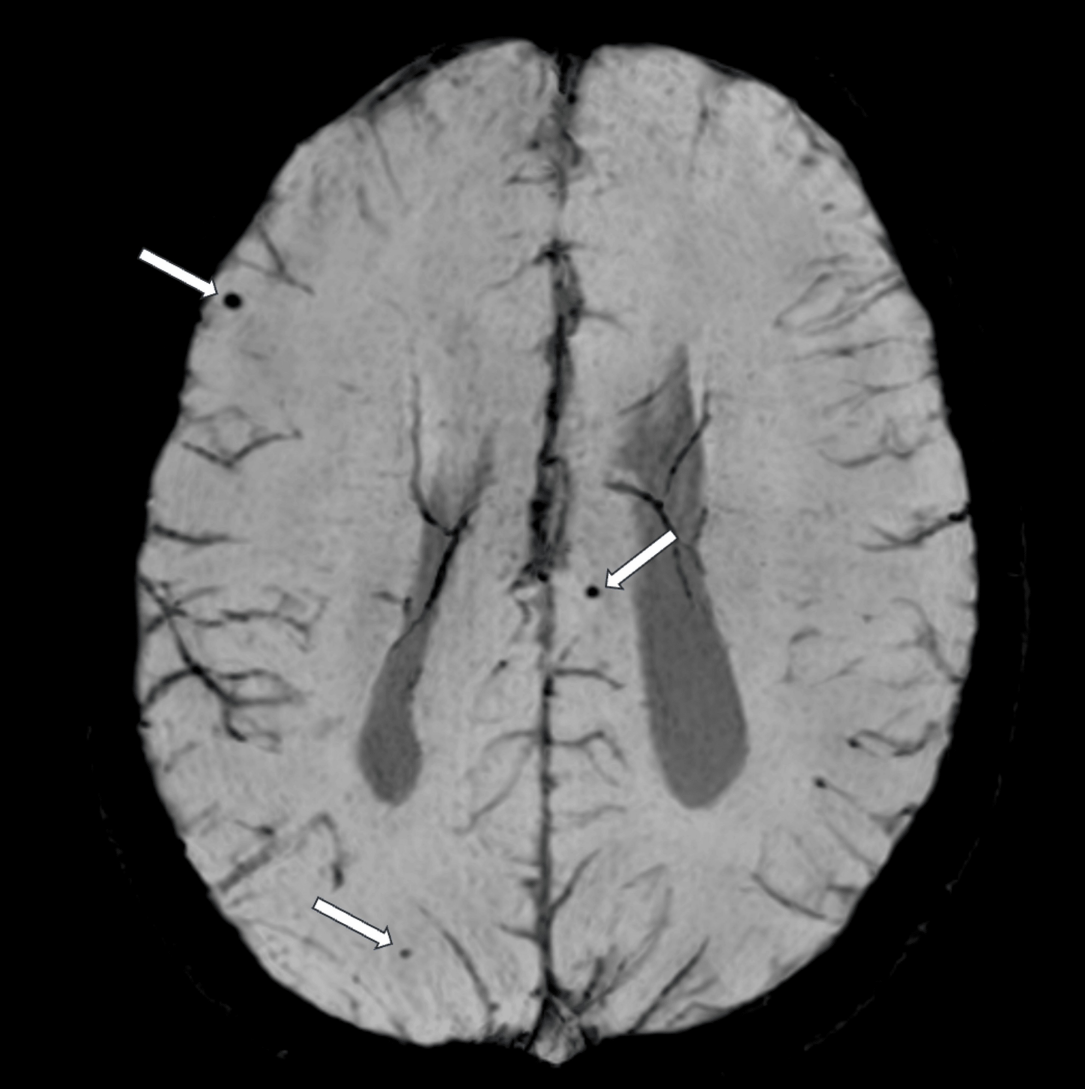
Axial-SWI MRI of the brain without contrast demonstrates punctate hemorrhages (white arrows) SWI - susceptibility-weighted imaging

At 13 years old, a multiplanar MRI of the brain without contrast was performed and demonstrated a dysplastic corpus callosum (Figure 2), colpocephaly with mild lateral and third ventriculomegaly (Figure 3), and an ovoid, dysmorphic appearance of the hippocampal formations (Figures 4-5) key distinctive features of Koolen-de Vries Syndrome [1,2,4]. This study also revealed nonspecific punctate foci of susceptibility in the bilateral frontal and temporal lobes, likely a punctate microhemorrhage presumably caused by cardiac abnormalities that required eventual surgical intervention (Figure 6). He continues to follow up with genetics and metabolism, developmental pediatrics, cardiology, pulmonology, ophthalmology, ENT, and orthopedic surgery.

## Discussion

Koolen de-Vries Syndrome (KdVS) is a rare genetic disorder with an autosomal dominant inheritance pattern caused by a 500- to 650-kilobase (kb) deletion at chromosome 17q21.31, including KANSL1 or a pathogenetic variant in KANSL1 [1-3]. Approximately 60% of KdVS cases result from heterozygous deletion, while the variant in KANSL1 accounts for 40% of cases [1]. KdVS can be identified in patients with developmental delay, neonatal/childhood hypotonia, congenital malformations, dysmorphic features of the skeleton, and behavioral symptoms [1-5]. Most individuals with KdVS experience mild-to-moderate intellectual disability alongside psychomotor and speech/language delays [1,2]. Children typically say their first words around the age of one; however, patients with KdVS say their first words between 2.5 and 3.5 years old, exemplifying the characteristic developmental delay [1]. 

The diagnosis of KdVS is established through molecular genetic testing and an assessment of family history [1]. If the typical phenotype is expressed, gene-targeted testing such as chromosomal microarray analysis (CMA), single-gene testing, or a multigene panel can be used for diagnosis [1]. However, it's crucial to note that comprehensive genomic testing becomes necessary when KdVS phenotypic traits are not evident. Additional forms of testing that can be considered include epigenetic signature analysis and karyotyping [1]. The prevalence of KdVS is unknown, though it is estimated that 17q21.31 deletion was found in 1:55,000 births [1,3]. Differential diagnosis encompasses other chromosomal deletions such as 22q11.2 deletion syndrome and other pathogenic variants such as lysine acetyltransferase 6B (KAT6B) and lysine acetyltransferase 8 (KAT8) [3]. KdVS can also be mistaken for Prader-Willi syndrome, Angelman syndrome, and cardiofaciocutaneous syndrome [1]. These differential diagnoses are based on similar findings of facial dysmorphic features, epilepsy, congenital heart defects, and other indications.

Manifestations of congenital heart defects, urogenital anomalies, and musculoskeletal abnormalities are present in 25-49% of patients; however, only 10-24% of individuals have hearing impairment [1,2]. Central nervous system anomalies, including epilepsy and brain malformations, are observed in approximately 80% of cases [1,3]. Less than 10% of affected individuals may also exhibit sacral dimples, dural ectasia, spina bifida, pineal cyst, and cervical spinal canal stenosis [1]. Patients may also present with cryptorchidism and skin-pigmentation abnormalities [1,4,5]. Neuroimaging typically reveals certain structural brain abnormalities, including ventriculomegaly, aplasia/hypoplasia of the corpus callosum, hydrocephalus, Chiari 2 malformation, intraventricular hemorrhage, and an ovoid hippocampus [1,2,4]. 

Our patient showed typical symptom onset along with lower incidence manifestations. The patient presented with an atrial spatial defect (ASD), early cryptorchidism, sensorineural hearing loss in the right ear, and idiopathic scoliosis. Brain MRI findings displayed in this case are examples of the neurodevelopmental features in neuroimaging for KdVS. The neuroimaging displayed a dysplastic corpus callosum, colpocephaly with mild lateral and third ventriculomegaly, and an ovoid, dysmorphic appearance of the hippocampal formations.

There is no cure for KdVS. Management primarily involves addressing its manifestations. This includes physiotherapy to address gross and fine motor delays, speech and feeding therapy, and educational programs tailored to manage intellectual disability. Regular monitoring is crucial, involving routine ophthalmic examinations for hypermetropia and strabismus, surveillance of spine deformities, and vigilance for potential complications affecting other organ systems [1]. GeneReview has listed the disease-specific supportive organizations that can potentially benefit individuals and their families inflicted with this disorder: Kool Kid Alliance and Koolen-de Vries Syndrome Foundation [1].

## Conclusions

KdVS is a low-incidence incurable disease caused by the deletion of 17q21.31 or a pathogenetic variant in KANSL1. Children affected have developmental delays and commonly a distinctive phenotype. This case demonstrated imaging of key distinctive features of Koolen-de Vries Syndrome, including scoliosis, a dysplastic corpus callosum, colpocephaly with mild lateral and third ventriculomegaly, and an ovoid, dysmorphic appearance of the hippocampal formations. In addition to these image findings, the patient also presented with an ASD, early cryptorchidism, and sensorineural hearing loss in the right ear, other manifestations commonly seen with KdVS. The case presented contributes to the medical community's knowledge of the neuroimaging characteristics and clinical presentation of KdVS.
